# Economic Cost of Suicide Among Culturally and Linguistically Diverse (CALD) Migrants in Australia

**DOI:** 10.3390/ijerph22060892

**Published:** 2025-06-03

**Authors:** Humaira Maheen, Christopher M. Doran

**Affiliations:** 1 Centre for Health Equity, Melbourne School of Population and Global Health, The University of Melbourne, Melbourne, VIC 3010, Australia; 2 Appleton and Manna Institutes, Central Queensland University, Brisbane, QLD 4000, Australia; c.doran@cqu.edu.au

**Keywords:** suicide, economic cost, CALD populations, mental health, Australian migrants

## Abstract

Background: Suicide and self-harm pose significant global public health challenges with substantial economic implications. Recent Australian evidence shows considerable variations in the prevalence of suicidal behaviours and mortality among culturally and ethnically diverse population groups. This study aims to estimate the associated economic cost of suicide among culturally and linguistically diverse (CALD) migrants in Australia. Methods: We evaluated the economic impact of suicide by considering the years of life lost, years of productive life lost, and overall economic costs, including direct, indirect, and intangible costs. We used data on suicide deaths in 2020 from the National Coronial Information System. Results: The estimated economic cost associated with 346 suicide deaths among CALD migrants is $2.9 billion (Australian dollars), with an average cost per fatality equivalent to $8.47 million. This estimate varies in the sensitivity analysis from $1.9 billion to $3.9 billion, depending on the average age of fatality, with corresponding average costs of $5.59 million to $11.35 million, respectively. These estimates do not capture costs associated with suicidal behaviours, which may substantially increase the economic burden. Conclusions: The significant economic impacts of CALD migrants’ suicide in Australia highlight the urgent need for a comprehensive national suicide prevention programme tailored for CALD migrant populations.

## 1. Introduction

Suicide is a significant public health concern, with more than 700,000 people dying by suicide every year worldwide [1]. The impact of suicide is substantial, with estimates suggesting that each death affects about seven people, including family members, colleagues, friends, and first responders [2]. Countries with high suicide rates bear a substantial economic burden, encompassing both direct costs (e.g., healthcare utilisation and emergency services) and indirect costs, such as lost productivity, as well as intangible costs associated with grief and psychological trauma among bereaved families and communities [3,4,5]. The economic cost of suicide, estimated at USD 53.1 billion in the United States (2015) [3], EUR 18.5 billion in France (2019) [4], and EUR 835 million in Ireland (2002) [5], highlights its substantial societal and economic impact.

In many Western countries where suicide is a significant public health issue, the extent of the problem within their migrant populations remains limited [6]. There is some knowledge about how certain migrant groups demonstrate a lower suicide risk compared to local-born populations, which is attributed to protective factors like cultural, familial, and religious influences [7,8]. However, emerging evidence demonstrates that some ethnic minority or migrant groups (Black Africans, Pacific Islanders, South Americans, and mixed-racial backgrounds) in high-income countries are disproportionately affected by suicide [9,10,11,12]. Critical factors that may explain suicidality among migrant populations include a history of trauma (particularly for refugees), assimilation into different cultures, the loss of cultural identity, and experiences of racism and discrimination in workplaces [8,13]. Despite the increasing recognition of suicide risks among ethnic minorities and migrant communities, comprehensive research on the related economic cost remains largely unexplored.

### 1.1. Culturally and Linguistically Diverse (CALD) Populations in Australia

Australia is one of the most multicultural countries in the world. Migrant populations are an integral part of Australia’s society, with approximately 30% of the population born overseas and 50% of them having at least one parent born overseas [14]. Within this broader migrant demographic, a significant proportion belong to what is commonly referred to in Australia as culturally and linguistically diverse (CALD) backgrounds. Individuals from CALD backgrounds typically include those born in non-English-speaking countries or those with parents from such countries. In the Australian context, migrants from non-English-speaking countries exclude individuals born in the United Kingdom, the United States, Canada, Ireland, New Zealand, and South Africa [14].

The term “CALD” does not refer to a single group but encompasses diverse populations distinguished by cultural, language, and religious identities [15]. While there are critiques of the term CALD as being overly broad and potentially obscuring important differences within and between groups [16], it nevertheless provides a useful framework, especially in public health research, for identifying population groups that need targeted support. The term remains widely used in Australian policies and programmes.

In Australia, the total economic cost of suicidal behaviour (including direct, indirect and intangible costs) was recently estimated to be in the range of AUD 30.5 billion in 2018 [17]. This cost is substantially high in some high-risk groups, e.g., young people (AUD 511 million in 2014) [18] or construction industry workers (AUD 3.97 billion in 2019) [17,19]. Recent Australian evidence on suicidal behaviours amongst migrant populations noted significant differences in the prevalence of suicidal thoughts, attempts, and mortality between groups [10,11], with some groups, like African, Pasifika, and South Americans, being particularly affected by suicide, yet the associated financial implications remain unknown. This paper aims to address this gap by providing an estimation of the economic cost of suicide among CALD migrants in Australia.

### 1.2. The Study

Using suicide mortality data for the year 2020 from the National Coronial Information System (NCIS), the study calculates the annual economic cost associated with suicide mortality for CALD migrant populations.

### 1.3. Ethics Approval

The study was conducted in accordance with the principles outlined in the Declaration of Helsinki. The study was approved by the Justice Human Research Ethics Committee, Department of Justice and Community Safety (reference, C.F./23/12049) and the Human Ethics Advisory Group (2022-23605-29220-3) at the University of Melbourne.

## 2. Materials and Methods

### 2.1. Data Sources

#### 2.1.1. Suicide Data

Data on intentional self-harm deaths were obtained from the NCIS, a web-based repository of coronial data from Australia and New Zealand [20]. The NCIS holds online records of coronial briefs created as part of investigations conducted by coroners into individual deaths. Restricted access to the NCIS web portal is granted to academics and researchers for research purposes through ethics approval. For this study, we obtained data on the age, sex, employment status, country of birth, and usual country of residence of individuals who died by suicide in 2020. This information was extracted only for “closed” cases with complete coronial records.

#### 2.1.2. Definition of CALD Migrants

For the purpose of this study, we used *country of birth*, *birthplace,* and *usual country of residence* as proxy indicators of CALD migrant populations. The NCIS uses the Standard Australian Classification of Countries (SACC) to code countries [21]. To identify cases from CALD backgrounds, we included those who were born in non-English-speaking countries, as defined by the Australian Bureau of Statistics (2013), and those for whom Australia was noted as their usual country of residence [14]. This excludes individuals born in Australia, Canada, New Zealand, the United States, South Africa, the UK, and Ireland. Using a country of birth or corresponding geographical region is considered an acceptable approach and is particularly useful for studying migrant populations [22], as it has also been employed in the peer-reviewed literature [7,10]. Table 1 outlines the inclusion and exclusion criteria used to identify individuals from CALD migrants for the study.

### 2.2. Data Analysis

The impact of suicide among CALD migrants was assessed using three measures: (1) years of life lost (YLL), (2) years of productive life lost (YPLL), and (3) the economic cost, which includes direct, indirect, and intangible costs. All costs reported in this section are in Australian dollars (2023). 

#### 2.2.1. Average Years of Life Lost (YLL)

The average age of a death by suicide was obtained from the NCIS. To calculate the average YLL per fatality, the average age of death by suicide is subtracted from the average life expectancy at birth, which is 85.8 years for females and 83.2 years for males [23]. Although we used the average age of death to calculate the average YLL per fatality, the average age of death varied in the sensitivity analysis.

#### 2.2.2. Years of Productive Life Lost (YPLL)

YPLL provides a proxy for the number of years of potential productive life (i.e., working-age potential) lost per fatality. The average YPLL is derived by subtracting the average age of a death by suicide (from NCIS) from the retirement age in Australia (66.5 years) [24]. As noted below, an adjustment is made to productivity estimates to allow for a proportion of the sample that may not be in the workforce. Nevertheless, the estimate of YPLL is an appropriate proxy for potential working life lost.

#### 2.2.3. Estimation of Economic Cost

A summary of the parameters used in this study to estimate the economic costs of CALD suicide is provided in Table 2. These parameters are used for estimating direct, indirect, or intangible costs (reported in Australian Dollars).

#### 2.2.4. Direct Cost

Direct cost in this analysis considers funeral costs and postvention bereavement costs. Although there are many other potential direct costs, such as medical, first responder or coronial inquiry costs, evidence suggests that these costs account for a small proportion of the total costs [17,18,25].

Funeral costs are estimated to be $10,411 [26]. It is acknowledged that funeral costs may vary depending on cultural or religious beliefs, so a conservative estimate is applied. Further, while funeral expenses may be associated with all deaths, fatality by suicide brings these costs forward. A fatality by suicide has a flow-on effect, with research suggesting that each fatality by suicide directly impacts between 6 and 135 people, including family members, work colleagues, friends, and first responders present at the time of death [2,27]. A conservative estimate is taken by assuming that the economic cost associated with suicide bereavement is $16,630 per person, multiplied by six people bereaved (adjusted to reflect AUD in 2023) [27,28].

#### 2.2.5. Indirect Cost

Employment status by sex was obtained from the NCIS. For those employed, lost economic productivity was calculated using the human capital method. This method considers the value of potential future earnings from the incident to the retirement age, assuming a discount profile and productivity loss. Average weekly earnings were estimated at $1672.70 and $1204.40 for males and females, respectively [29]. It is assumed that those who were unemployed received unemployment benefits, equivalent to an average allowance of $816.90 per fortnight [30]. For those unemployed, the future income stream represents a saving to the government. A long-term productivity factor of 1.2% per annum reflects growth in income/welfare [31]. This figure is used in conjunction with a discount profile that includes a discount rate of 3.84% (the average over the period 1990–2022) [32] adjusted for an inflation rate of 2.4% (the average over the period 1990–2022) [33] to determine the present value of future income streams. Also included in the indirect cost category is the loss of government revenue through taxation and deadweight losses associated with the administration of taxation and welfare payments. The loss of government revenue reflects the tax losses due to foregone income and is valued using the marginal tax rate appropriate to average weekly earnings (i.e., 32.5%) [34]. Deadweight costs due to inefficiencies incurred through tax losses are estimated at 10.81% of the total net present value of lost government revenue (i.e., tax revenue). Deadweight costs due to inefficiencies incurred by social welfare payments are estimated to be 9.75% of the total net present value of welfare payments [35].

**Table 2:** Summary of key cost parameters.

Parameter	Male	Female	Source
Average weekly earnings	$1673	$1204	Australian Bureau of Statistics [29]
Marginal tax rate of average earnings	32.5%	32.5%	Australian Taxation Office [33]
Proportion employed	32.2%	24.3%	National Coronial Information System [20]
Proportion unemployed	16.5%	16.4%	National Coronial Information System [20]
Average life expectancy at birth (years)	83.2	85.8	United Nations [23]
Average retirement age (years)	66.5	66.5	Australian Government Department of Social Services [24]
Average age of fatality (years)	52.9	50.5	National Coronial Information System [20]
Average years of life lost	30.2	35.3	Calculated
Average productive years lost	13.6	16.0	Calculated
Discount rate (per annum)	3.84%	3.84%	Reserve Bank of Australia [31]
Inflation rate (per annum)	2.4%	2.4%	Macrotrends [32]
Productivity rate (per annum)	1.20%	1.20%	Australian Government Treasury [30]
Average unemployment benefit (yearly)	$21,239	$21,239	Australian Government Services Australia [29]
Average funeral expenses	$10,411	$10,411	White Lady Funerals [26]
Postvention costs—suicide bereavement (family and friends)	$99,780	$99,780	Comans et al. [27], Australian Bureau of Statistics
Deadweight loss of government revenue	10.81%	10.81%	Safe Work Australia [34]
Deadweight costs of welfare payments	9.75%	9.75%	Safe Work Australia [34]
Value of a statistical life	$5,766,695	$5,766,695	Office of Best Practice Regulation [36]
Value of a statistical life year	$250,675	$250,675	Office of Best Practice Regulation [36]

#### 2.2.6. Intangible Cost

Intangible costs relate to the community value of a lost life, which is estimated using a “willingness to pay” approach based on the value of a statistical life. As noted in the Productivity Commission report [17], the Bureau of Transport Infrastructure and Regional Economics [36] uses this approach to calculate the costs associated with road fatalities. The value of a statistical life is an estimate of the financial value society places on reducing or avoiding the death of one person. By convention, this is assumed to be based on a healthy person living for another 40 years. It is known as a “statistical” life because it is not the life of any particular person. An estimate of the value of life is, therefore, a tool for decision-making, not the value placed on any particular person. There are a variety of methods used to value life, but the “willingness to pay” method is viewed as the most appropriate technique [37]. Unlike other methods, such as the human capital model, which captures the discounted value of future earnings, the willingness to pay method quantifies non-market preferences and values, such as quality of life, health, and leisure [38]. The Office of Best Practice Regulation [37] has estimated the value of a statistical life year to be $250,675 million, adjusted to 2023 dollars.

#### 2.2.7. Sensitivity Analyses

In accordance with good modelling practice, a number of sensitivity analyses were undertaken to test the robustness of the results to variations in key parameters [39]. The discount rate varied from 3.84% to a low of 0% and high of 5%; the average male weekly earnings varied from $1673 by ±20% ($1338–$2007); average female weekly earnings varied from 1204 by ±20% ($964–$1445); the value of a statistical life year varied from $250,675 by ±20% ($200,540–$300,810); the average age of male fatality varied from 52.9 years (42.3–63.5 years); and the average age of female fatality varied from 50.5 years (40.4–60.6 years). Allowing for variations in the average age of fatality impacts both the average years of life lost and the average productive years of life lost. This, in turn, impacts both productivity and statistical life year estimates.

## 3. Results

Table 3 provides an overview of CALD suicides in Australia in 2020. A total of 346 CALD Australians aged 15 and above died by suicide in 2020. Similar to the general population, male suicide was higher than female suicide, with 70% of suicide deaths being males. Women’s mean age of suicide was slightly lower than that of men. The age-standardised suicide rates of males were 11.6 per 100,000 and females were 5.1 per 100,000.

Table 4 provides an overview of the economic cost for the CALD population. Using data from the table, the average cost per fatality is $8.04 million and $9.23 million for males and females, respectively. Combining the average cost with the number of fatalities results in a total economic cost associated with CALD suicide of $2932 million $2.93 billion each year. Applying the value of a statistical life year to the total number of potential years lost is the key cost driver in cost estimates, accounting for 94% of all costs. Although the average cost is higher for females than males, the total cost for males is estimated at $1977 million compared with $923 million for females, driven by the larger number of suicides in males.

### Sensitivity Analysis

Table 5 provides an overview of the sensitivity analysis results. A key parameter driving the results is the average fatality rate. Varying the mean age of the male and female fatality by ±20% results in a range from $1.94 billion to $3.93 billion (compared with $2.93 billion using the base case average age). As noted earlier, modifying the average age of fatality impacts the potential and productive years of life lost. The younger the age of fatality, the higher the value of indirect and indirect costs. Varying the age of fatality results in a range of average costs per fatality from $5.59 million (older age) to $11.35 million (younger age). Modifying the value of a statistical life year is also a key cost driver of economic results. Changes to the discount rate and average weekly earnings change indirect cost estimation.

## 4. Discussion

The estimated economic cost associated with 346 suicide deaths among CALD migrants is $2.9 billion (in 2023 Australian dollars), with the average cost per fatality equivalent to $8.47 million. This estimate varies in the sensitivity analysis from $1.9 billion to $3.9 billion, depending on the average age of fatality, with corresponding average costs of $5.59 million to $11.35 million, respectively. These results are consistent with population estimates generated by the Australian Government Productivity Commission [17], at an average cost of $9.4 million per fatality in 2018.

Our study is the first to demonstrate the economic impact of suicide among diverse populations, not only in Australia but worldwide, where no such evidence exists for migrants or ethnic minorities. Given the significant economic burden associated with suicide among CALD migrant populations, there is a strong rationale for developing a comprehensive, culturally responsive national suicide prevention programme. The limited global evidence on migrant suicide recognises the importance of integrating diverse cultural interpretations of suicide, coping mechanisms [40], and their associated help-seeking practices into prevention efforts [41,42]. These interpretations significantly shape when and how individuals engage with mental health services, which, in turn, can influence the effectiveness and economic impact of interventions. For instance, while the availability of interpreters at health services is crucial to improving the quality of service delivery, it may be insufficient in overcoming the barriers to seeking care in the first place [43,44]. This is particularly relevant for suicidal behaviours, which are often subject to multiple layers of stigma in some culturally diverse communities [43,45]. The stigma may relate to mental illness, suicide itself, and the act of seeking help. Addressing these complexities is crucial—not only for preventing suicide but also for reducing the broader economic burden associated with suicide among migrant populations.

Globally, suicide prevention is predominantly influenced by a biomedical lens, with mental health services being the primary response to suicide. Typically, these programmes are designed for the general population and later adapted, with some language-related adjustments for migrants. In Australia, CALD migrant groups engage with mental health services later and less frequently [46], often due to the stigma of mental health and suicide or negative experiences with mental health professionals [47], commonly viewing these services as culturally misaligned [48]. There have been some programmes and projects on suicide prevention that target CALD populations in Australia. Notable programmes include the translation of suicide prevention resources into multiple languages [49], translation and interpretation services at the Primary Health Network’s commissioned mental health services [50], and programmes for trauma victims from refugee backgrounds [51]. There are also short-term projects, including gatekeeper training programmes for faith leaders and other community members, training culturally diverse media outlets on responsible reporting of suicide incidents [52] and training for Services Australia personnel [53]. While these initiatives are significant, most of them are one-off projects with no follow-up. The longer-term programmes for CALD migrant populations largely address language barriers to accessing mental health services [49]. Language is just one part of cultural responsiveness, so while necessary, these initiatives do not address the varied cultural perspectives on suicide and help-seeking, which diminishes the cultural responsiveness of these efforts.

The recent National Suicide Prevention Strategy (2025–2035) [54] in Australia recognised the importance of addressing the social and structural determinants of distress as part of effective suicide prevention. It also identifies some CALD groups as priority populations, presenting a timely opportunity to increase investment in culturally responsive suicide prevention initiatives. Our study highlights the significant economic implications of continued inaction and provides an evidence base to inform more targeted, effective, and culturally appropriate suicide prevention programmes for migrants from CALD backgrounds.

### 4.1. Limitations

#### 4.1.1. Suicide Data Limitations

This study focuses on migrant populations. It utilised data from the NCIS [20], which reports only the country of birth, birthplace, and the usual country of residence of individuals to indicate migration status. The NCIS does not collect data on migration indicators, which could lead to an underestimation of the true representation of CALD migrants and the misclassification of individuals (for example, Australians born in non-English-speaking countries). We want to note that in this study, CALD migrants did not include migrant descendants, also known as second-generation migrants (those born in Australia to migrant parents from CALD backgrounds). Furthermore, visa or citizenship status was also not included in the NCIS data, which, to some extent, may affect the estimated cost (an underestimate most likely), given that those under Australian citizenship or permanent residency (e.g., working holiday makers) may have different working rights, tax rates, and eligibility for unemployment benefits.

Despite these limitations, the country of birth remains a pivotal proxy indicator of migrant status and is widely employed in peer-reviewed literature and government reports [15,55]. In Australia, given that previous migration waves have been linked to countries of origin (such as migration from the UK, China, Europe, Asia, and the Middle East), the country of birth provides a useful context for understanding migration. This study acknowledges the limitations of its findings due to the absence of migration and ethnicity indicators in the data and advocates for the integration of such variables in the NCIS and other population datasets.

#### 4.1.2. Post-Pandemic Study

It is important to note that 2020 was immediately after the onset of the COVID-19 pandemic. Fortunately, suicide rates in Australia remained unchanged during the pandemic, with slightly fewer suicide deaths reported compared to other years [56]; therefore, this should not affect the interpretation of our findings.

#### 4.1.3. Suicidal Behaviours Are Not Included

It is also noteworthy that these estimates do not capture costs associated with suicidal ideation, thoughts, or self-harm attempts. Recent data from the Australian National Study suggests that for Australians aged 16–85 years, 16.7% had thoughts of suicide, 7.7% had made plans to attempt suicide, and 4.8% had attempted suicide in their lifetime [28]. As noted by Kinchin et al. [18], self-harming behaviour also results in a range of costs, including the loss of potential years of life and productive years of life. However, it is difficult to ascertain suicidal behaviour data from national surveys, given that some of these surveys are not very inclusive for CALD migrants with limited language proficiency and temporary visa status [57]. In this context, the NCIS offers a comprehensive and consistent source of suicide data for migrant populations.

#### 4.1.4. Costing Methodology

Authors of costing studies note that economic costing is not an exact science, and due to various limitations in data and methods, various assumptions are required [18,58,59]. As noted by the Productivity Commission [17], their estimates of the economic cost of suicidal behaviour are considered conservative. For example, their estimates exclude government expenditure directly on suicide prevention activities. The Australian Government spent almost $69 million on suicide prevention under its National Suicide Prevention Program in 2025 [54]. State and Territory Governments also fund their suicide prevention activities designed to meet local needs. However, this expenditure is currently not being publicly reported or consistently consolidated.

## 5. Conclusions

For the year 2020, the economic cost associated with CALD migrant suicide is $2.9 billion (in 2023 Australian dollars). This study highlights the significant economic impacts of suicide among CALD migrant populations and emphasises the need for a comprehensive national suicide prevention programme tailored to diverse communities. Effective engagement with CALD communities is crucial for designing these initiatives to understand how suicide manifests in different cultures, how help is sought, and which coping mechanisms are successful. Such engagement will enable the development of more efficient suicide prevention strategies, ultimately leading to a reduction in the economic costs associated with suicide and self-harm behaviours.

## Figures and Tables

**Table 1:** Inclusion and exclusion criteria.

SACC Major Group	Excluded Countries of Origin	Included Countries of Origin
1 Oceania and Antarctica	Australia and New Zealand	Rest of the countries from the region
2 North-West Europe	All countries	
3 Southern and Eastern Europe		All countries
4 North Africa and the Middle East		All countries
5 South-East Asia		All countries
6 North-East Asia		All countries
7 Southern and Central Asia		All countries
8 Americas	Excluding US and Canada	Rest of the countries from the region
9 Sub-Saharan Africa	Excluding South Africa	Rest of the countries from the region

**Table 3:** Profile of CALD suicide (15 and above) in Australia, 2020.

Variable	CALD Status	
	Male (n = 246)	Female (n = 100)
Age-standardised rate (per 100,000) with 95% confidence interval	11.6 (9.9, 13.3)	5.1 (4.0, 6.1)
Mean, SD age of suicide	52.9, 20.1	50.5, 19.4
Employment status at the time of death		
Employed	32.2%	24.3%
Unemployed	16.5%	16.4%
Not in the labour force	40.2%	44.1%
Unlikely to be known	11.2%	15.2%

**Table 4:** Economic cost breakdown of CALD population in Australia (reported in AUD).

	Male	Female	Total
CALD suicide deaths—2020	246	100	346
Potential years of life lost	7441	3534	11,355
Potential years of productive life lost	3340	1600	5116
Direct cost			
Total funeral expenses	$2,561,212	$1,041,143	$3,602,355
Total postvention costs	$24,545,880	$9,978,000	$34,523,880
*Subtotal direct costs*	*$27,107,092*	*$11,019,143*	*$38,126,235*
Indirect costs			
Net present value of lost income for those employed (excluding taxation revenue lost)	$64,274,815	$24,003,979	$119,225,928
Net present value of taxation foregone	$30,947,133	$7,801,293	$38,748,427
	Male	Female	Total
Net present value of deadweight loss due to reduced government taxation	$3,345,385	$843,320	$4,188,705
Net present value of JobSeeker payments saved for those unemployed	−$11,888,326	−$5,490,109	−$17,378,435
Net present value of deadweight gains due to fewer JobSeeker payments	−$1,159,112	−$535,286	−$1,694,397
*Subtotal indirect costs*	*$85,519,895*	*$26,623,198*	*$143,090,227*
Intangible costs			
Value of a statistical life year	$250,675	$250,675	$250,675
Total value of life lost	$1,865,322,543	$885,769,399	$2,751,091,943
*Subtotal intangible costs*	*$1,865,322,543*	*$885,769,399*	*$2,751,091,943*
TOTAL ECONOMIC COST	$1,977,949,530	$923,411,740	$2,932,308,404

**Table 5:** Economic cost sensitivity analysis *(reported in AUD)*.

		Change to Discount Rate		Change to AWE		Change to VSLY		Change to Age of Fatality	
	Base Case Estimate	Low	High	Low	High	Low	High	Low	High
CALD suicide deaths—2020	346	346	346	346	346	346	346	346	346
Potential years of life lost	11,355	11,355	11,355	11,355	11,355	11,355	11,355	14,933	7776
Potential years of productive life lost	5116	5116	5116	5116	5116	5116	5116	8695	1538
Direct cost									
Total funeral expenses	$3,602,355	$3,602,355	$3,602,355	$3,602,355	$3,602,355	$3,602,355	$3,602,355	$3,602,355	$3,602,355
Total postvention costs	$34,523,880	$34,523,880	$34,523,880	$34,523,880	$34,523,880	$34,523,880	$34,523,880	$34,523,880	$34,523,880
*Subtotal direct costs*	*$38,126,235*	*$38,126,235*	*$38,126,235*	*$38,126,235*	*$38,126,235*	*$38,126,235*	*$38,126,235*	*$38,126,235*	*$38,126,235*
Indirect costs									
Net present value of lost income for those employed (excluding taxation revenue lost)	$119,225,928	$156,239,193	$110,642,726	$95,380,742	$143,071,113	$119,225,928	$119,225,928	$198,820,799	$44,630,835
Net present value of taxation foregone	$38,748,427	$50,777,738	$35,958,886	$30,998,741	$46,498,112	$38,748,427	$38,748,427	$58,550,659	$14,505,021
Net present value of deadweight loss due to reduced government taxation	$4,188,705	$5,489,073	$3,887,156	$3,350,964	$5,026,446	$4,188,705	$4,188,705	$6,329,326	$1,567,993
Net present value of JobSeeker payments for those unemployed	−$17,378,435	−$22,884,304	−$16,107,194	−$17,378,435	−$17,378,435	−$17,378,435	−$17,378,435	−$28,683,967	−$8,065,343
Net present value of deadweight gains due to JobSeeker payments	−$1,694,397	−$2,231,220	−$1,570,451	−$1,694,397	−$1,694,397	−$1,694,397	−$1,694,397	−$2,796,687	−$786,371
*Subtotal indirect costs*	*$143,090,227*	*$187,390,480*	*$132,811,122*	*$110,657,615*	*$175,522,839*	*$143,090,227*	*$143,090,227*	*$232,220,130*	*$51,852,135*
Intangible costs									
Value of a statistical life year	$250,675	$250,675	$250,675	$250,675	$250,675	$250,675	$300,810	$250,675	$250,675
Total value of life lost	$2,751,091,943	$2,751,091,943	$2,751,091,943	$2,751,091,943	$2,751,091,943	$2,378,027,434	$3,301,310,331	$3,656,983,841	$1,845,200,044
*Subtotal intangible costs*	*$2,751,091,943*	*$2,751,091,943*	*$2,751,091,943*	*$2,751,091,943*	*$2,751,091,943*	*$2,378,027,434*	*$3,301,310,331*	*$3,656,983,841*	*$1,845,200,044*
TOTAL ECONOMIC COST	$2,932,308,404	$2,976,608,658	$2,922,029,299	$2,899,875,792	$2,964,741,016	$2,559,243,896	$3,482,526,793	$3,927,330,206	$1,935,178,415

## Data Availability

The datasets presented in this article are not readily available because of confidentiality reasons. Requests to access the datasets should be directed to the National Coronial Information System Office.
